# A case report of an endoscopic approach to life‐threatening cecal variceal hemorrhage

**DOI:** 10.1002/jgh3.12733

**Published:** 2022-04-07

**Authors:** Kirsty E MacFarlane, Nicholas J Fischer

**Affiliations:** ^1^ Auckland Hospital Auckland District Health Board Auckland New Zealand; ^2^ Department of surgery Basingstoke and North Hampshire Hospitals Basingstoke UK

**Keywords:** cecal varices, endoscopic glue injection, hemorrhage

## Abstract

Cecal varices are a rare cause of gastrointestinal bleeding in patients with cirrhosis. We describe a 29‐year‐old man with decompensated alcoholic cirrhosis who developed gastrointestinal bleeding in the hospital. A computed tomography mesenteric angiogram showed bleeding cecal varices, which were successfully treated by glue injection therapy at colonoscopy. The procedure appeared to be complicated by bacteremia due to *Escherichia coli*.

## Graphical abstract

Cecal varices have a high mortality rate likely due to being amongst the rarest complications of portosystemic hypertension. This is the case report of a 29‐year‐old man who developed life threatening hemorrhaging cecal varices but was treated successfully and survived, bringing to the academic world some potential treatment options and adding to the paucity of literature.

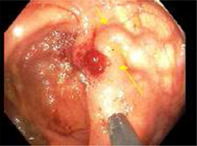

## Introduction

Bleeding varices associated with cirrhosis typically occur in the distal esophagus. However, varices may bleed at other sites, particularly the stomach and duodenum, in approximately 5% of patients.^1^, ^2^ Bleeding from cecal varices is rare.^1^ We present a case report of life‐threatening cecal variceal hemorrhage, which was successfully treated with endoscopic glue injection but complicated by *Escherichia coli* sepsis and subsequently treated with intravenous antibiotics.

## Case report

Varices typically occur in the distal esophagus but may occur anywhere in the gastrointestinal tract. Non‐esophageal variceal hemorrhages account for approximately 5% of all bleeding varices.^1^, ^2^ Cecal varices are among the rarest non‐esophageal varices.^1^, ^3^, ^4^

We report a case of a 29‐year‐old man with decompensated alcoholic liver cirrhosis complicated by life‐threatening hemorrhage from varices in the cecum. This man initially presented with abdominal distension and bleeding gums, on a background of alcoholic liver cirrhosis, without melena or hematemesis. He self‐reported drinking 20 units of ethanol per day for the last 10 years with minimal abstinent periods. He had no other significant medical background.

His clinical assessment revealed jaundice, ascites, poor dentition, and muscle wasting, consistent with alcoholic malnutrition. Investigations were consistent with decompensated liver disease with a serum bilirubin of 174 μmol/L, albumin 14 g/L, prothrombin ratio 1.8, sodium 132 mmol/L, hemoglobin 87 g/L, and platelet count 105 × 10^9^/L.

**Figure 1:**
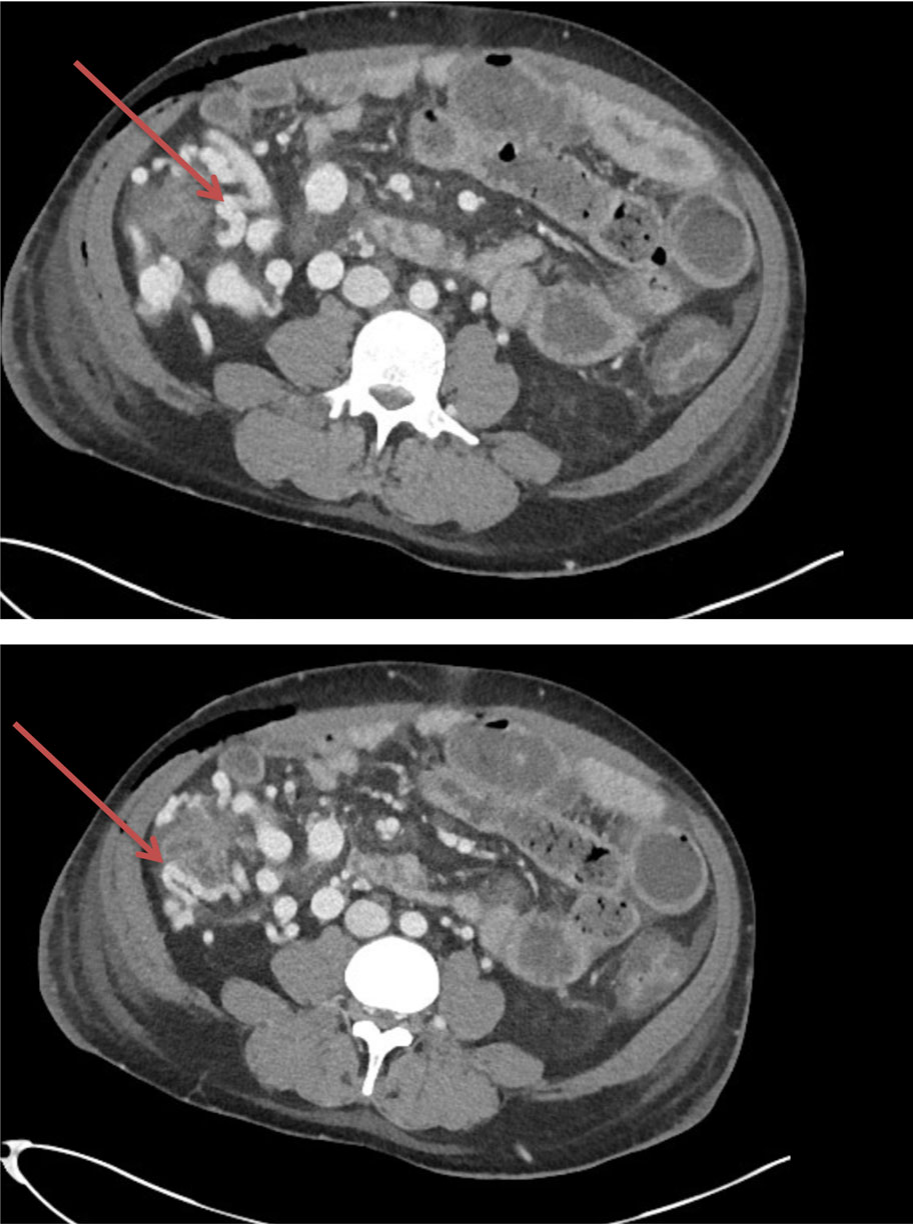
Computed tomography mesenteric angiogram showing cecal varices.

Initial management consisted of abdominal paracentesis with albumin replacement and alcohol withdrawal monitoring. On his third day of admission, he developed overt rectal bleeding. Clinical examination did not find any anorectal abnormality. He became hypotensive and tachycardic with an associated hemoglobin fall to 46 g/L. He was resuscitated with blood products and taken for an urgent upper gastrointestinal endoscopy under general anesthesia, which did not find a source of bleeding. He remained hemodynamically unstable with tachycardia and hypotension. He proceeded to have a computed tomography mesenteric angiogram (CTMA), which showed large submucosal cecal varices with features suggestive of recent hemorrhage (Fig. 1).

Interventional radiology specialists were consulted but intervention was not considered technically possible after due consideration. Given his severe decompensated liver failure and portal hypertension, he was not considered a candidate for surgery. Therefore, endoscopic management was the only option. He continued to actively bleed and was resuscitated with 10 units of red blood cells, 7 units of fresh frozen plasma, 4 units of cryoprecipitate, and 3 units of platelets.

**Figure 2:**
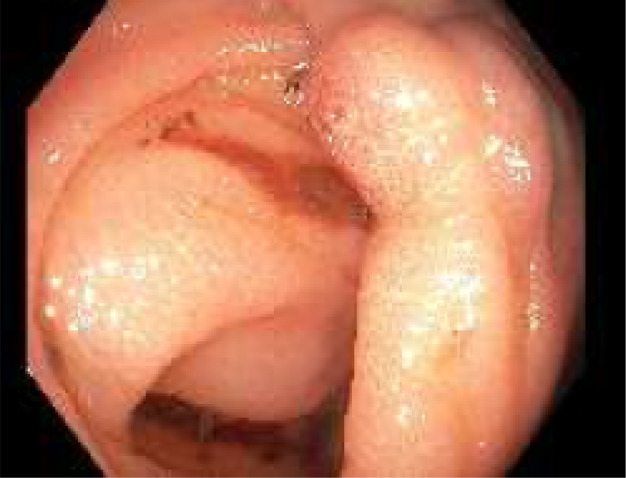
Colonoscopy showing cecal varix hemorrhage.

**Figure 3:**
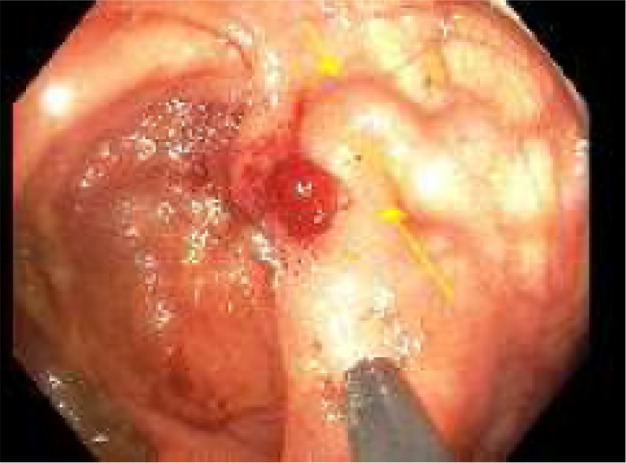
Cecal varix after the first injection of 0.5‐mL Histoacryl glue mixed with Lipiodel.

**Figure 4:**
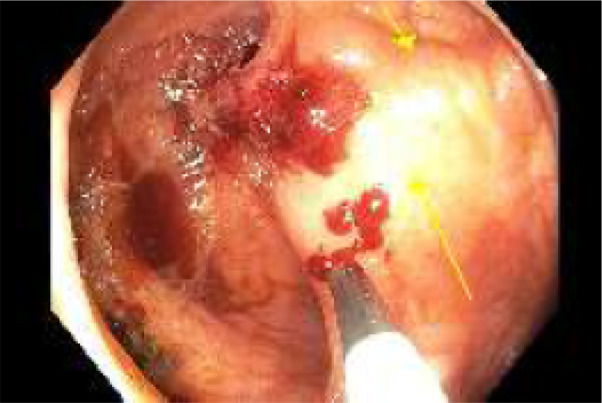
Cecal varix after three 0.5‐mL injections of Histoacryl glue mixed with Lipiodel.

A bowel preparation (Picoprep) was administered via a nasogastric tube in the intensive care unit. Colonoscopy was performed. The large varix in the cecum was located with stigmata of recent hemorrhage (Fig. 2). The varix was successfully injected with three injections of 0.5 ml of Histoacryl glue mixed with Lipiodel (Figs 3, 4).

Hemodynamic stability was achieved with no further bleeding. He remained in hospital for two more days and was discharged home with alcohol cessation support. He re‐presented 4 days later, however, systemically unwell with fevers but no localizing symptoms to aid diagnosis. Blood cultures, diagnostic paracentesis, urine analysis, chest radiograph, and CT of the abdomen and pelvis were carried out. There was no clot propagation beyond the cecal varix and no intraabdominal abscess seen on the CT. Blood cultures subsequently revealed *E. coli*. The working diagnosis was the colonization of the glue complex in the now thrombosed varices in the cecum, presumably introduced at the time of injection or secondary to translocation of enteric organisms. A 6‐week course of intravenous cefuroxime was administered. He remained well at clinical review 2 months later with no further episodes of fever or bleeding.

## Discussion

This case report highlights the diagnostic and management dilemma of hemorrhage from cecal varices. It is a rare complication of portal hypertension, with only 5% of reported variceal bleeding to be non‐esophageal; of these, only 14% are colonic.^1^

Management of bleeding cecal varices is not well defined, as there are no prospective trials due to its rarity. A multidisciplinary approach is advised; however, options may be limited depending on the expertise available. Gastroscopy should be performed in the first instance, and if negative, it should be followed by contrast enhanced CT.

Treatment options can be broadly grouped into radiologic, surgical, and endoscopic. While these hemostatic treatments are only temporizing measures, they may allow the patient time to recompensate and potentially become a candidate for transjugular intrahepatic portosystemic shunts (TIPS) or liver transplantation to address the underlying portal hypertension.

Interventional radiologic approaches such as direct percutaneous embolization (both anterograde and retrograde) and portosytemic decompression such as a TIPS should be considered; however, patients such as in this case report may not be well enough to tolerate this in the acute setting. Furthermore, decompensated liver disease with hepatic enceophalopathy is a contraindication to TIPS.^2^ Balloon‐occluded retrograde transvenous obliteration (B‐RTO) has gained attraction for gastric varices, with some papers reporting 89% cessation in bleeding; however, as the portal pressure is not addressed with this approach, there is often an increase in bleeding in esophageal varices and at other sites.^1^, ^2^ Surgery is usually not pursued because of the high morbidity and mortality risk in liver decompensation; however, resection has been reported as a salvage procedure.^2^, ^5^

Endoscopic treatment includes sclerotherapy with glue or thrombin injection, or band ligation. Barriers to endoscopic intervention include the feasibility of adequate bowel preparation in an unstable patient and the availability of experienced interventional endoscopists. For large (>2 cm) gastric varices, evidence for injection *versus* banding suggests that banding may have higher rates of re‐bleeding. However, whether this evidence is applicable to the treatment of cecal varices is unknown.^2^, ^5^, ^6^ Propagation of thrombus from a cecal varix into the main superior mesenteric vein or portal vein is a recognized potential complication of glue or thrombin injection. In this case, however, we report *E. coli* septicemia as a probable complication of endoscopic glue injection. This complication has clinical significance in its own right, as we could not find any other cases reporting this syndrome in the literature. Therefore, due consideration to antimicrobial prophylaxis to cover enteric organisms before endoscopic injection seems appropriate.

In conclusion, endoscopic glue injection for the treatment of life‐threatening hemorrhage from cecal varices in an unstable patient can be successful. Systemic infection secondary to this treatment may occur, and broad spectrum antibiotics should be considered at the time.
